# Indents

**Published:** 1907-05-15

**Authors:** 


### INDENTS.

**☞ Brief Articles of Technical or Scientific Value will receive notice and comment. Contributions invited.**

**Care of the Handpiece.** Disconnect the handpiece and dip it in a jar of alcohol containing 10 per cent eucalyptus oil. That is the best solution for the purpose.—L. M. Markham, *Dental Brief.*

**Amalgam Repairs.** Fresh amalgam can be made to adhere to an old amalgam filling by merely coating the freshly exposed surface of the latter with chlorhydric acid. The same object can be attained by treating the freshly exposed surface with silver nitrate.—*Dental Brief.*

**Pressure Anesthesia and Cataphoresis Identical.** I have had considerable experience with cataphoresis for desensitizing the dentine, and the results obtained from the use of the high pressure syringe are identical as far as my observation goes. C. C. Patton, Boston, Mass.

**Chloral Hydrate.** Apply a crystal of Chloral Hydrate to an aching pulp and cover with soft gutta-percha to stop the pain. For Pericementitis, make a solution of one part Chloral Hydrate in five parts of glycerine and paint over the parts involved.—Dr. C. L. Quincerot, in January, 1907, *Cosmos.*

**Upper Impressions.** If it is found difficult to remove an impression for full superior denture, have the patient close the lips and *blow* with sufficient force to distend the cheeks and the impression will drop down, no matter how tight it may have been.—R. C. Traynham, *Practical Dental Journal.*

**Cleaning Files.** When a vulcanite file becomes clogged with rubber and plaster, it may be easily cleaned by wrapping absorbent cotton around it and saturate the cotton with chloroform. In about ten minutes it can be cleaned perfectly by the use of a stiff brush wheel on the lathe.—V. P. Perisho, in December *Review.*

**A Hot Brick.** A common brick is the best thing to warm rubber on before packing. It holds heat a long time and the rubber will not stick to it. Don't get a smooth one, just a common red brick, and you will wonder why such a good thing was not found out before.—O. F. Brigham, *American Dental Journal.*

**Sterilization of Dentures.** Sulfurous acid will absolutely deodorize and disinfect a denture, and not merely cover the odor of a plate that has been worn in the mouth. Place a few drops in a little water and immerse the case in the solution at night and cleanse with soap and brush in the morning.—J. KENNERLY, *British Dental Journal.*

**Fatalities.** A man of Bluffton, O., expired in a dentist's chair, November 6, while under the influence of chloroform.—November 1, a man in Coshocton, N. Y., died from heart disease while in the dental chair.—October 23, a man in Saskatoon, Sask, Canada, died in the dental chair while under the influence of ethyl chlorid. *Digest.*

**To Clean Wax.** After melting and straining boil the the wax for 20 minutes in a solution composed of half-ounce oxalic acid to each quart of water. Old wax may be made cleaner than new. Two drops of oil of cassia added to each pound keeps wax aseptic and renders it less unpleasant to the patient.—Thos. Fletcher, *Pacific Medical Journal.*

**Extracting Abscessed Teeth.** I have found by experience that the extraction of abscessed teeth is not a dangerous operation if the oral cavity is swabbed with an antiseptic wash before and after. Always take precaution after the tooth is out to give the socket a thorough cleansing. This is where many operators make their mistakes.—*Odontoblast.*

